# Social Support for Improved ART Adherence and Retention in Care among Older People Living with HIV in Urban South Africa: A Complex Balance between Disclosure and Stigma

**DOI:** 10.3390/ijerph191811473

**Published:** 2022-09-12

**Authors:** Lucia Knight, Enid Schatz

**Affiliations:** 1 Division of Social & Behavioural Sciences, School of Public Health, University of Cape Town, Rondebosch, Cape Town 7701, South Africa; 2 Department of Public Health, University of Missouri, Columbia, MO 65211, USA

**Keywords:** older people living with HIV (OPLWH), ART adherence, social support, disclosure, stigma, HIV competence

## Abstract

The number of older people living with HIV (OPLWH) (aged 50-plus) in South Africa is increasing as people age with HIV or are newly infected. OPLWH are potentially vulnerable because of the intersection of age-related and HIV stigmas, co-morbidities, and lack of social support. Evidence from younger populations suggests that social support can improve ART adherence and retention in care. Further, HIV status disclosure plays a role in mediating social support and may reduce stigma by facilitating access to social support. This paper draws on qualitative research with OPLWH to explore the complex associations between disclosure, social support, and HIV stigma among OPLWH in urban Western Cape. The findings demonstrate that OPLWH receive most of their support from their family and this support can facilitate adherence to ART and retention in care. However, social support is facilitated by participants’ disclosure, thus, when perceived stigma limits disclosure, social support is less accessible. Gender, age, and pre-existing vulnerability also affect disclosure to and support from kin and community. Given that social support, particularly from family members, amplifies HIV care access and ART adherence, encouraging disclosure stimulating household HIV competency is likely to both address anticipated stigma and support improved OPLWH’s health outcomes.

## 1. Introduction

Evidence from low- and middle-income countries (LMIC) shows the valuable role that social support plays to reduce stigma and increase adherence to antiretroviral treatment (ART) for younger people living with HIV. However, we know comparatively little about these issues for older populations [1,2]. Despite the rising numbers of people aged 50 and above living with HIV in sub-Saharan Africa [3], few studies exist that provide insight into how ART access and HIV care for older Africans might be facilitated by social support, defined as assistance or care received to help alleviate or respond to stressors. We aim to begin to fill this gap.

Of the 37.7 million people living with HIV globally in 2020, over half (20.6 million) live in Eastern and Southern Africa [4]. In 2017, 12% (7% females and 5% males) of the 7.9 million people living with HIV in South Africa were above 50 years of age [5]. Recent evidence suggests that along with the increasing rates of HIV in older people, access to ART has resulted in people aging with HIV, resulting in a large number of older people living with HIV (OPLWH) [6]. This group is potentially vulnerable as a result of the intersection of age-related and HIV stigmas, co-morbidities with non-communicable diseases (NCDs) and also, importantly, may be socially isolated or lack social support [7,8].

The large number of OPLWH in South Africa make it essential to examine the relationship between social support and living well with HIV for this age group. In other contexts and age-groups (younger adults), there is evidence that social support facilitates positive health outcomes by improving ART adherence and retention in care [2,9]. HIV status disclosure plays a role in mediating social support and may reduce stigma, in ways that can ensure access to social support [2,10,11]. Whereas those unable to disclose are potentially at risk of increased social isolation [12,13], those who have disclosed often report lower levels of internalized stigma [1]. However, there are risks associated with disclosure including increased visibility that can trigger other types of stigma [14,15,16]. Among older persons who often experience increased dependence on their social network as a result of aging, the complex associations between disclosure, social support, and HIV stigma need to be documented. In this paper we focus on social support among OPLWH in urban South Africa, and the ways that disclosure influences social support and moderates stigma.

Research suggests that membership of social networks is important for people living with HIV (PLWH). A social network—relationships that give support, identity and a sense of belonging to an individual—includes family, friends and those in the community and may facilitate social support for those living with HIV. Those with more diverse networks may have better outcomes because of they have broader sources on which to draw [17]. Social networks as a source of social support to PLWH have been demonstrated in various studies with evidence from South Africa in particular highlighting the significant role that extended family networks and social norms around family obligation play in providing social support [16,18,19,20,21,22]. Similarly, work on older South Africans’ social networks also suggests the importance of family [23,24,25]. It is important to note that in the South African context ‘family’ often means extended kin who can live in the same or other physical households and are linked by financial and care arrangements, not just immediate or nuclear families [20,26]. The evidence for the social support for OPLWH within LMICs that are under-resourced, such as South Africa, however, is limited as the bulk of the research and evidence to date is focused on those of reproductive ages.

Social support has been characterized in numerous ways and includes a range of types of support. We summarise the aspects of support under three primary categories: emotional support, instrumental support and informational support received from people within one’s social network [23,24,25,27,28]. Emotional support includes empathy, love, trust, and caring, whereas instrumental support is material or financial and therefore more practical support and informational support is characterized by the provision of information that people can use to assist them [29].

In high-income settings there is evidence that, for older persons living with HIV, social support is critical to help meet their emotional and functional needs [30]. In these contexts, family, partners, friends, and social groups were all considered as sources of strength and facilitated coping and resilience [31,32]. Those older people with social support, particularly practical and emotional support, often have better medication adherence outcomes [27,28,33]. Documented barriers to social support for OPLWH in high income countries and contexts include personal issues such as: lack of disclosure, a desire to be self-reliant and independent, and not wanting to be a burden [34,35]. Community or social network barriers include: stigma and fear about HIV, ageism, no family for social support, and deaths within the community from AIDS [12]. These barriers could explain the greater social isolation observed among OPLWH compared with their HIV-negative counterparts [36]. Thus, social isolation may increase in the context of illness-related distress [37] and may significantly impact retention in care and adherence behaviour among OPLWH [13,28,38,39]. Stigma has been found to decrease social support among OPLWH [40]; however, when robust social support exists, it may facilitate resilience and decrease the stigma among OPLWH, therefore improving health outcomes [16,31].

The relationship between social support and stigma is often facilitated by disclosure of the HIV positive person’s status to those within their social network [16]. Disclosure which has been shown to be important in the South African context where health care workers encourage those who are tested, to both bring along someone to support them in their initiation on ART (treatment buddy) and, also, encourage disclosure to a family member. These may be important ways to facilitate access to social support [11,41,42]. Resistance or hesitancy to disclose is often linked to and influenced by the risk of stigma or discrimination which may lead to reduced social support [15,16,43]. The interactions between stigma and disclosure and the role they play in the ability of older people to secure and benefit from social support in this context have not been explored to date and require additional research.

In their exploration of the differences in social support between younger and older PLWH in the United States, Emlet et al. [13] draw on the convoy theory of social support to frame their findings. The convoy is the social network of those who are able to provide social support at any given time, family, friends and community, which is influenced by the current circumstances, events and people surrounding, in this case, the OPLWH [13]. This conceptual framework, defined originally by Kahn and Antonucci [44], suggests that social support changes over time and is dependent on the specific needs and realities of those who require it, in this case older people living with HIV. The framework suggests that the ability to secure social support depends on what the authors call the properties of the person, which incorporates the individual’s demographic characteristics including their age and gender but, in this case, also their relationship status and their living situation, and also the properties of their situation, which includes their own role expectation, resources and demands. Our previous research has shown that stage of life course, gender and age all have had a role to play in the decision to, and testing experience of OPLWH [45]. It therefore makes sense that these characteristics are likely to impact on the social support of these people too [46].

Although the evidence for the role of social support for PLWH is growing, there is a dearth of evidence from resource constrained, LMIC countries and contexts, such as South Africa, for data about the role that social support plays in the health outcomes of OPLWH. We examine the role of disclosure and stigma and how these interact with, facilitate or are potential barriers to social support within an older population in urban South Africa, to begin to fill this gap.

## 2. Materials and Methods

These data are from two urban communities on the outskirts of Cape Town—Langa and Khayelitsha—situated on the ‘Cape Flats’. Their populations are predominantly black, have low socio-economic status and rates of unemployment are high [47]. Infrastructure varies but access to basic services is generally poor and the communities have a mix of formal and informal housing settlements [47]. Despite this relatively poor infrastructure and widespread poverty, both communities have relatively good access to health services, social and developmental non-governmental organizations, community initiatives and social development and welfare services [48].

In this study, conducted in 2016–2017, we employed a descriptive qualitative study design to explore OPLWH’s social support and reported health implications [49]. Participants who were HIV positive and over 50 years old were purposively sampled for maximum variation for both age and gender. Two recruitment strategies identified study participants: a survey list from a local NCD research project, with a self-report HIV status question, and convenience sampling at clinics. Having multiple recruitment strategies enabled both the enrolment of men and those at older ages with HIV, groups that are often hard to reach or find. Participants were recruited by the qualitative interviewer either within the community or within the clinic depending on the approach. Interviews then took place either within the participants home or in a quiet and private space within the clinic.

All respondents provided informed consent prior to the study; interviews were audio-recorded with permission. isiXhosa data were transcribed and translated into English by the interviewer. The University of the Western Cape Bio-medical Research Ethics Committee provided ethical approval and the Western Cape Provincial Department of Health permission for access to facilities.

All data were collected using semi-structured qualitative interviews that took between one and one and a half hours to complete. The interviews were conducted in isiXhosa by a trained and experienced qualitative interviewer, herself an older person as defined within the study, which was one of the ways we facilitated rapport with respondents. The interviews with OPLWH explored their experiences of living with HIV, including social support, disclosure, and stigma.

Data analysis was coded and managed using NVivo Pro version 11.0 and led by LK in consultation with the research team (ES and the qualitative interviewer). Following familiarization, data were coded iteratively and inductively using a thematic approach [50]. Codes were reorganized to develop themes and sub-themes with reference to, and in alignment with, the theoretical framework. We then analysed the themes to assess patterns within and across groups by age and gender, as well as in relation to one another, e.g., how narratives about disclosure connected to social support, how these were different depending on whether the disclosure was to family or the wider community, and how social support was associated with adherence.

## 3. Findings

Our findings are based on a final sample that included 23 respondents of whom 13 were from Khayelitsha, 10 were women, and 13 were between 50 and 60 years of age. None of the female participants sampled were in a regular relationship and almost all of the men were married or partnered (see Table 1 for more details). All of the respondents’ first languages were isiXhosa. These OPLWH reported that family was by far the most significant source of all types of support; broader social networks of friends, neighbours, and other members of the community were considered a secondary, although often quite complicated, source of support. The results relating to these key sources of support will address various relevant themes that emerged from the data and are informed by the key conceptual issues identified as part of the convoy theory described above. Therefore, we first discuss the negotiations and decision-making processes involved to secure social support or the decision not to, as influenced by disclosure and the reality of stigma. We then focus on the types of support OPLWH are able to secure and how social support facilitates adherence and retention in care. As gender and stage of life are key factors that influence the above processes related to disclosure, stigma, support choice, the nature of social support, and their influence on ART adherence [46], we note differences that emerged among our participants where relevant.

### 3.1. Disclosure as a Facilitator of Social Support

Disclosure served as an important facilitator of support for our respondents, whether the disclosure was active and purposeful, or at times accidental. Disclosure allowed respondents to get assistance that facilitated ART use—reminders to take medication, assistance in getting to/from clinic appointments—and emotional support to not feel badly about their status.

#### 3.1.1. Families Accept, “No One Asks to Be Sick”

Close family relationships were an important source of social support for OPLWH in our sample, but disclosure was necessary to initiate this social support. Despite hesitancy and worry noted by some respondents, disclosure was actively chosen to facilitate getting support from the people with whom they lived or had close interpersonal relationships, or from those for whom they felt a strong affiliation. As one man noted:

*R: I found it was difficult [when I found out I was HIV positive], because in my family [my status is] known by my sister only, the one that comes after me [younger], by me telling her; it wasn’t easy because I was shocked, plus it is known by my wife and my children*

*I: In the family what made you choose your sister, what caused you to tell her?*

*R: My sister and I support each other.*

*(male, 64 years, married with HIV-positive wife)*

In other cases, disclosure to family was something that respondents’ felt some encouragement from their health care providers to do as part of the counselling process. Therefore, disclosure was again undertaken actively with the explicit intention of getting support for their ART adherence. As one widow explained, “*It [HIV status] is only known here at home. The nurses [say] they [my family] must know because I might become sick, they must know what is what [HIV status].*” *(female, 62 years, widow)*

Disclosure was sometimes unintentional, even if one’s status was not actively hidden. In such cases, health concerns, the need to go to the clinic or hospital, or observation of medication led to questions and subsequent disclosure. One male respondent reported only disclosing his status to his mother a few weeks before the interview, despite being diagnosed in 2008. He described his fears of disclosure and how his eventual disclosure occurred:

*…I didn’t want to tell [my mother] because she was going to go around these houses [neighbourhood] crying and talking about [my HIV status] with other people, that’s why I said to the counsellor when asked whom I trust then I chose my sister… [My mother] wanted to see the pills and asked what the pills are for and then I said they are for HIV and she gave them back [to me].*

*(male, 50 years, single)*

Despite his fear, his mother was calm and accepting in her reaction, perhaps due to a greater acceptance of those living with HIV between his initial diagnosis in 2008 and his disclosure to her nearly a decade later.

In general, our respondents spoke about disclosure to those they were close to—either in terms of relationship or proximity—as fairly straightforward and without much risk or fear of stigma or discrimination. Thus, though some were hesitant, their disclosure was met with acceptance:

*[My family] felt hurt but they said they have to accept [my status] because if not that will make me sick, so we must feel free the same way in which you are about it and I said ‘my children you must accept this, I’ve also accepted it because I did not invite the illness, nobody asks to get sick’ … No, they don’t even have a tendency, because I’m a sick person, to detest me. They are still treating me well.*

*(female, 60 years, single)*

Even where they had concerns, they were usually pleasantly surprised by their family’s response: sharing their status did not change the way they were treated, and in fact in many cases facilitated accessing support.

It is notable within the findings that gender and age are important factors in terms of familial networks and also impact on disclosure, but also the availability of family to provide support. This is largely due to stage of life that people within our study find themselves in. All except one of the men in the study in their 50s and early 60s were married or in long-term co-habiting partnerships and all of those except for one had partners who were also HIV positive. In the one instance where the partner was negative, she knew about her partner’s status and was supportive. This close relationship with a partner who knows and shares one’s status was important for the men in the sample. In addition, men often also lived with children who varied in age but in some cases were still school aged. Male respondents who lived in nuclear or multi-generational households largely received good social support from within their household from their nuclear or co-resident family members. Women in the sample on the other hand were not in cohabiting partnerships and although they reported family support and disclosure it tended to be more instrumental and less emotional support resulting from disclosure to those within their family networks. This seemed to influence men and women’s different attitudes to and their need for support outside of the family as will be discussed below.

#### 3.1.2. Community Disclosure Is More Complicated

Disclosure to the community was less straightforward and less uniform across our sample. Whereas many of the respondents did note that people who knew them very well or lived close to them knew their HIV status, the examples of active and purposeful disclosure to the wider community were reported much less frequently than such disclosures to family. In many cases disclosure beyond the family was unintentional and not necessarily chosen, as in the example below:

*No, there is a neighbour of mine who was employed here at the clinic, we would meet here in the clinic, she used to work here in Ubuntu Centre and has now been transferred to the other side now, she knows about it in that way.*

*(female, 61 years, widow)*

However, in this case, the inadvertent disclosure ended up being positive and a source of important support for this individual who herself could rely on limited familial support, as indicated in the way the respondent reframes this unintentional disclosure as active and a means for opening discussions.

*…there’s no-one that I disclosed to, they will know about it from mere observation [seeing her at the clinic], and a neighbour that is close to me who whenever I’m not well or need Panado [paracetamol] I go to her because she was also working at the HIV [clinic] and would say to me she has three HIV positive children and therefore I must not worry because if a person eats your treatment there is no problem, we normally chat together…I had told her because she’s a neighbour and we would also see each other here at the clinic and I wouldn’t be ashamed when I see her because I had disclosed to her.*

*(female, 61 years, widow)*

In only a few cases was disclosure to those in the community something actively chosen. The examples of where people did choose to disclose to friends or neighbours were often where they had very good close and supportive relationships with those disclosed to and where they were less able to draw on reliable familial support:

*Things are very good for me, nobody rejects me, I’m not someone who is isolated, I can relate well with other people, I mix with them and laugh with them; whenever people need help they think of coming to me, I help them and they give me money; at times I observe that a person is having a hard life like yesterday I was visiting a friend of mine who is occasionally [not well treated] …we know [neighbours and friends in the community] about each other [HIV status] we just talk about it and laugh it off… I am very happy, we [close neighbours] help each other, if one needs anything you can get it from your neighbour by calling out for assistance; even one who has children and needs to go to work will leave her children in our care.*

*(female, 64 years, separated)*

Alternatively, there were a few examples where people were working in or active members of the community and felt it was important to disclose either for the benefit of the community or because it was an obligation linked to their activism around HIV. As indicated below:

*Some just hear [about his HIV status] when I chat and the advice I give them is that they must be careful of it and I try and give them that counselling that was given to me…It is important that they know [about his HIV status] so that in order to seek advice they can approach you and ask how you managed to withstand it because if you hide it though you need not be proud of it but observe the situation and say brothers if a person could have HIV he must ask us who already have it as to what it entails.*

*(male, +−55–60 years, married with HIV-negative wife)*

Disclosure more broadly than the family was often driven by insufficient family support necessitating additional support from outside. Men in the sample were more likely than women to be able to rely on family, notably their spouse, and thus often lacked the impetus to disclose to or seek support from beyond the family. This is likely linked to their slightly different life stage—slightly younger and less likely to be widowed—and their ability to rely heavily on the support of their spouse/partner or close nuclear family. It is clear from the results above that women, who were much more likely in our sample to be single or widowed and without direct family support, more commonly reported disclosure to those outside of the household and were also less likely to disclose as a result of fear (see below).

#### 3.1.3. Fear of Stigma despite Potential Promise of Support

Men, in particular, were reluctant to share their status beyond their families. This may also be closely linked to fear of stigma and discrimination or just feeling that the status of the individual is private and a belief that their status only needs to be known within the family. This is demonstrated in the below quote:

*No at work I never told them, if I were to tell them I could lose my job or they would talk about it and yet that’s not right, it’s yours and your family…it’s my problem, it’s not theirs and I also don’t ask them with regard to themselves; sometimes at other places they would want the doctor to write down what you are suffering from; I’ve never said the doctor must write, I just told them I’ve got TB…even in the church I never told them…I have a reason, this has to be my secret because they also have their own illnesses which I don’t know about, and it’s my secret and my family’s, it’s yours and theirs only, I don’t see the need for anybody else to know even if it’s a friend, I don’t trust a friend anyway, he might talk about it to other people.*

*(male, 56 years, married)*

As in the example above, fear of stigma connects with hesitancy to disclose status more broadly than the family. Another respondent also described his fear of disclosure:

*…I hear the things said by people about other people who have [HIV] that are known to them… they [talk about those people] in a bad way…Perhaps they say…in the case of those who default treatment…can’t you see how thin he is, he eats those pills but then I’m confused in that you see another person having gained weight and they say “he has gained weight because of these pills “and then you feel that you don‘t know what they actually want, you understand. Then I decided to keep my mouth shut as long as I’m known to my family at home.*

*(Male, +−50 years, partner HIV-positive)*

The lack of disclosure may also have been linked to people outside of the family not being willing and or able to provide the required social support. One woman highlighted there being little point in disclosing because it would not result in support:

*There’s no-one else that I can say I’m looking to [for support]. People in this community…we just talk but not in a way that they can perhaps help you with something. I would describe myself as not being alone because there are people, that I stay with here in the community. There is nothing they can help me with although they are here.*

*(female, 55 years, single)*

Although disclosure to family was an important means of facilitating support, the lower likelihood of receiving support from community members (among women) as well as a fear of stigma and discrimination (among men) limited broader disclosure, and thus also limited sources of potential support.

### 3.2. Having Social Support Leads to Better Adherence

We highlight the ways in which our respondents talked about three primary categories of social support (emotional, instrumental, and informational) that individuals might receive from those in their social network [27,28,29] and how this supported their ART adherence. In many cases it is difficult to untangle these types of support—reminders to take ART are both instrumental as they provide practical support, but also emotional as they are an exhibition of love and caring. Once disclosure occurred, extended family became a key source of support in ways that directly and indirectly impacted ART adherence and retention in HIV care. At times, particularly when individuals had little in the way of familial support, OPLWH’s social support, namely in the forms of practical and information support, came from neighbours, friends, and clinicians, as well.

#### 3.2.1. Blending Emotional and Instrumental Support

Despite resistance and many of the respondents not wishing to disclose their status beyond their family units, there were also clear examples where disclosure to neighbours or friends was beneficial and resulted in emotional and instrumental support. These included examples of practical assistance, linked to affective ties and the closeness and quality of the relationship.

*I am happy here because the people living in my community are warm people, we help each other, even when I started becoming ill, they used to be very helpful. They brought me food and things that I needed.*

*(male, +−55–60 years, married with HIV-negative wife)*

Within families, similar efforts by family members made respondents feel well cared for, as well as facilitating ART adherence:

*Even my grandchild he would ask “father have you already taken your pills?” and also “mother have you already taken your pills?” and when I say he should give me my pills and then he will ask “should I also give [to] mother?”*

*(male, 67 years, married with HIV-positive wife)*

This direct support through practical reminders to take ART was most commonly reported to be provided by those who are living in close proximity—children, grandchildren, or spouses. These reminders were experienced as emotional support as well as practical support, as they exhibited love and care that motivated the reminders.

*I said, my wife, the best is for you to take your treatment in order to be right and for you to live a long life because if you break off and drink, as soon as you do not drink even for two days, the virus will be increasing inside you and yet if you drink continuously it can decrease. My wife and I, we are the same, she is open.*

*(male, 57 years, married with HIV-positive wife)*

In this instance a male OPLWH and his wife were both positive and on ART and providing each other with support. However, many of our respondents lived in multi-generational households and the individual family member who provided the support varied.

*I am being cared for in all ways and my daughter reminds me even if I am in bed and would say “father, father, here are your pills…father, father have you eaten already” and then I would ask her to bring my pills to drink.*

*(male, +−50 years, married with HIV-positive wife)*

Another participant, age 54 with a wife who is also HIV-positive, talked about his children and wife getting up in the morning to “*prepare porridge for me, after that they give me water to drink my pills.*”. Preparing food and drink to accompany taking treatment assisted in adherence but also connotes love and caretaking, reminding the OPLWH to take their treatment but also ensuring that people were eating and staying healthy.

Family members providing assistance with picking up medication from the facility was another form of instrumental support that facilitated good adherence to treatment,

*When I am very ill my children come and fetch [medication package] on my behalf.*

*(female, 71 years, widow)*

Other forms of support provided to OPLWH by their family were in-kind or financial assistance that indirectly facilitated ART adherence or retention in care or supported the health and well-being of the OPLWH. A few of the older people provided examples of their family members either providing them with the money for or actual transport to the facility for their ART pick-ups or to attend the facility.

Although current commonly prescribed ART medication no longer needs to be taken with food, the messaging about taking treatment accompanied by food seems to be prevalent among health care providers and may be justified by this being a good point of reminder for taking regular treatment.

*Yes, even when I have to go to Tygerberg one of [my sons] because two of them have cars, will transport me and will phone me while there to find out how far [I am and if] I have been attended to and then when [I’m] finished I’m fetched again.*

*(female, 61 years, widow)*

A few of the respondents also mentioned getting either money or groceries from their family members—both those who lived within their household and not—that meant they could eat and be healthy, which they characterised as important support.

*[My son who lives away and works] would say “no don’t buy groceries just pay the other things, I’ll do the groceries and other things and I do buy clothes. It’s just at certain times because his mother is alive, and the person I live with is not his mother, so he comes at intervals and then goes to live with his mother again…I tell him to buy items and not give me money.*

*(male, 61 years, married with HIV-positive partner)*

As mentioned above, emotional support, care, and encouragement that may accompany more practical support are also essential to ART adherence, health, and retention in care. Respondents talked about the care and support that took the form of presence or reassurance as in the instance below where a respondent was in hospital.

*[they support me] very much and even when I was sick, they were here when I was sick last year, they all came out here and checked on me at the hospital; I was admitted for a week and the second week I was discharged.*

*(male, 56 years, married)*

Other family members also provided more general care and support in terms of encouragement and supportive words as in the quote below:

*My sister told me this is a health problem that is found in people and that I should not be thinking that it is me alone, I must withstand it because I will also get better just like other people.*

*(female, 59, widow)*

As with the example above, presence was an important concept along with the idea that people’s love, affection, and care did not change despite the change in the person’s HIV status.

*No, there is not a single thing that I saw has changed [in my relationship with my family]. My family loves me very much as I said, most of them came [to my house] when I was sick.*

*(female, 55, single)*

This was noted by many of the respondents who felt that the lack of change in relationships was an important indicator of their families’ acceptance of both them and their status.

#### 3.2.2. Missing Support

Whereas many respondents outlined ways in which they received support from family, those whom they lived and those to whom they are related to or feel a strong affiliation, family was not always as supportive as might be hoped. A few OPLWH, particularly women who were more likely to be single or widowed than men, provided examples of family who despite being related and in some cases living within very close proximity to them were not supportive or actually made OPLWH feel rejected in some way.

*R:…she’s [her daughter] here in Cape Town*

*I: Does she also not have a contribution?*

*R: No. When we pass each other on the road then sometimes I feel as if I can kill myself, we don’t greet each other, I don’t know what I have done to her, I have been asking her all these years, I don’t know… I don’t know [what the cause of the rift is] she doesn’t explain.*

*(female, 58 years, single)*

The lack of contribution or support in the example above seems rooted in an existing problem in the relationship between mother and daughter and not likely to be something that has resulted from her HIV status but impacts her access to support nonetheless. Another example was provided by a woman who explained that although one of her children was supportive, her son who lived with her may not even know about her HIV status.

*They know [about HIV status] especially my daughter; it could be that my son doesn’t know because he doesn’t care about anything…but the other [children] know about it.*

*(female, 62 years, widow)*

As above, this example also seems to be linked to an existing strained or fractured relationship where the women’s son is not involved in her life and therefore cannot be counted on to care for her or even show interest.

Whereas family support was sometimes missing, it was interesting how little informational support was mentioned in comparison with other types of social support. One of the few examples of family members assisting by providing informational support was of a daughter who helped her mother to understand her prescription cards and juggling medications for both HIV and other conditions,

*No, and the cards are separate, and I do not feel challenged, and my child also helps me about how to take them and where to put the different pills for the different [illnesses].*

*(female, 60 years, single)*

Most of our respondents were contending with a complexity of appointments and treatments for the multiple chronic conditions [51]. Some OPLWH also held onto false beliefs and continued to profess erroneous information, e.g., that they must take their ART with food despite this no longer being a requirement [52]. Thus, informational support is something that appears to be missing.

## 4. Discussion

Understanding the available social support for OPLWH reveals (a) the important people within their social network that could be pivotal to improve their retention in care and adherence to medication, and (b) the direct links between disclosure and support within families and in the broader community. Our study showed that OPLWH rely on their networks, particularly family members, for social support. Emotional, instrumental, and to a lesser degree informational, support received had both direct and indirect impacts on adherence to ART and retention in care. This is in line with findings from other studies with OPLWH that show that family and relatives are an important source of various types of social support [12,53], and that PLWH in South Africa value the social support they receive from family [16,20].

Findings from high-income countries suggest that family support may be less important than that of friends for OPLWH [13,31,34,54]. The experience of aging in South Africa is quite different from these contexts, however, as is the nature of the HIV epidemic (heterosexual and generalised). There is evidence from South Africa that HIV disclosure is a way that individuals secure social support from family [10,15,16,43]. Among our sample, disclosure facilitated social support from family. The way disclosure came about differed across participants though. For some it was active and purposeful in order to secure social support, for others health care workers encouraged and supported the OPLWH to disclose to their families, for some it was unintentional. Previous analyses of these data also show the importance of social support from family as a key means of improving acceptability of ART adherence, retention in care, and reducing stigma among respondents [55].

Social support from family took all forms but was mostly emotional and instrumental. The OPLWH described both direct and indirect ways in which the social support they were able to secure and received assisted them with their adherence to ART and retention in care. It is notable that other findings from this data highlight some of the challenges that OPLWH face. OPLWH experience food insecurity [52]. Barriers to accessing HIV care include distance and the need to access multiple facilities or appointments as a result of co-morbidities or disability or illness [51,55]. In this analysis, we show that familial support was important for the OPLWH in our study to overcome these challenges. Familial support and its value is not necessarily unique to this population, as our previous research has shown, the challenges with access, compounded by co-morbidity and limits to physical ability are possibly more strongly felt and experienced by those who are older [51,52,55].

Maman and colleagues [16] explained that whereas disclosing one’s HIV status to the family could lead to an increase in social support, disclosure could also attract negative social outcomes including stigma, discrimination, and violence. This is clearly reflected in the feelings of our respondents and was a concern in their decision to disclose to family. It is notable that none of the respondents spoke specifically about fearing stigma related to their HIV as a result of their age, although this has been documented in some other research in terms of treatment by providers [56]. Other findings from this dataset suggest that some of the OPLWH in this study may have not been tested for HIV as a result of being older and assumed to no longer be sexually active or at risk of HIV, suggesting potential bias [45]. In general, despite fears or concerns about disclosure to family most people who did choose to disclose to family members were met with relatively positive responses, some of which they were surprised by and others which were expected as people had close relationships.

Despite almost all of the respondents highlighting some sort of social support from family that aided, either directly or indirectly, in their ability to adhere to treatment or retention in care, as with most social relationships, the intra-familial relationships were not without complexity and conflict. As noted in previous work conducted by the first author, family relationships are a constant negotiation based on a complex but culturally powerful interplay of obligation to family, affective ties or love, and the need to reciprocate [20]. Examples in the data show that where relationships were already strained or there was existing conflict it was harder to rely on family members for support and or care.

Other HIV positive people within the family or household were an important factor for disclosure, as people felt more comfortable to disclose because HIV was already a topic of conversation/known to the members of the household. They were also a source of mutual support with a number of the respondents talking about giving or receiving support with other family members. This links with the notion of HIV competent households as proposed by Masquillier et al. [10,22] where the social environment in which people live is supportive and conducive to adherence and retention in care. It is important to note that mental health may be another factor impacting the relationship between social support and adherence—leading to loss in following up HIV care or discontinuing ART. DiGennaro and colleagues found very high prevalence of mental health disorders among youth living with HIV—nearly three-quarters reported a mental health disorder (e.g., anxiety, depression, PTSD, or drug or alcohol misuse) compared with 35% among youth not living with HIV [57]. Stigma and mental health are closely related, as are psychosocial support and social support, DiGennaro and colleagues highlight the importance of mental health in its relation to HIV, stigma, and ART adherence, and suggest the need for integration of mental health services into general health services [57,58]. Although not a focus of this paper, such services would likely support OPLWH who are experiencing stigma and worry.

The convoy theory of social support denotes that life stage, in this case measured by age, along with gender and social situation are important determinants of access to social support [13]. This appears to hold true for our sample. We see marked differences by gender related to life stage and circumstance, and how gender affected disclosure to family and subsequent social support. Although family was important regardless of gender, the men in our sample seemed to rely more heavily on close emotional and day-to-day practical support from their spouses and partners than the women within the sample. These differences are not surprising, and previous data show that gender plays a role in HIV testing and diagnosis, such that men in our sample were more likely to be married or in co-habiting partnerships with spouses or partners who influenced their decision to get tested [45].

The OPLWH in our sample were not hugely reliant on social support outside of their close family or household. Disclosure beyond kin was much more complex and was heavily influenced by both anticipated (perceived future bias or mistreatment resulting from others knowing one’s status) and community-level stigma (operating at a community level) that resulted in a fear of people outside of a trusted circle of confidants knowing one’s status [14]. In some cases, this was linked to a sense of privacy—a perception that those outside of the family did not need to know because it was not their business—whereas for others it was clearly linked to a tangible fear of losing one’s job if an employer found out, or being talked about badly or in a judgemental way by community members. The fear of disclosure to co-workers or employers has been widely noted elsewhere [27] as has anticipated stigma and it’s role on preventing disclosure [15,16,41,54]. In some cases, the decision not to disclose was a more pragmatic one linked to a perception that people in the community were not able to provide much in the way of tangible support and so there was no perceived benefit from disclosure. There were a few notable exceptions where people did mention disclosing and benefiting from this disclosure to friends and others outside of the family.

As with the gendered differences discussed above, it is interesting to note that women, who often had fewer options to obtain support from close family, reported more disclosure and receiving informational or emotional support from those outside of the family. Men also seemed to have greater resistance to, and fear of, disclosure more broadly. This may have been linked to men’s lack of need for external support, as they were more able to secure the social support they needed within the family. However, it may have also been due to anticipated stigma they were concerned about encountering outside of the household. South African research shows that men are more likely than women to be impacted by anticipated stigma and that this influences their decision to take up HIV testing [59]. It is possible that this perceived stigma is further heightened because of age resulting in increased intersectional stigma, although this was not something that the respondents noted in their responses [54].

One of the most interesting findings in this research suggests that being older and at an increased risk of age-related illness, disability, and need to access health care was an important reason that people did not feel the need to disclose their HIV status. For younger people, illness, sudden weight loss or gain, need to access health care and to take treatment likely require an explanation in ways not true for older persons. Older persons in this community also suffer from very high rates of both TB and NCDs. In many cases, OPLWH were able to request support for health issues from people within the community without a need to disclose or discuss their HIV status. Instead, they drew on their good relationships within the community, and their status as older and generally unwell.

Based on our study findings, OPLWH draw largely from family as their primary source of informal social support, receiving a variety of types of support. This understanding could also inform the design and implementation of interventions that might encourage or facilitate this form of social support [60]. Public health interventions could be designed around these sources to improve the quality of support provided to OPLWH. For instance, interventions should seek to improve households’ or families HIV competence and encourage disclosure, which as our results show, is a key first step for OPLWH to be able to access social support. This may be particularly important in terms of increasing competence around providing informational support—about how and when to take ART—but also in combination with other medications given the high levels of multimorbidity in this population, which was not reported to the same extent as emotional and instrumental support. Household HIV competency would ensure that older people are able to live and be cared for in an environment in which the patient can be supported across the HIV care continuum in a sustainable manner [10,22]. This should be an environment where OPLWH can: (1) gain, share, and translate HIV-related knowledge to enhance prevention and treatment behaviour; (2) feel safe to disclose their HIV status and engage in dialogue about HIV; (3) take ownership of their HIV condition and responsibility for safe sexual practices, testing, and ART; (4) feel supported to adhere to ART and remain in care, as well as manage the medication and care for their co-morbidities; and (5) be receptive to outside support [22].

### Limitations

The findings of this study are based on a relatively small sample of OPLWH, though they still represent a range of ages and genders of OPLWH engaged in HIV care and treatment from two urban communities in Cape Town. The results are not generalisable and do not consider the realities of OPLWH outside of the health system or those outside of this geographical setting. Our sample of men were on average younger than the sample of women, which may have affected their stage in life and experience of social support and decision to disclose.

## 5. Conclusions

We explored the sources and benefits of social support among OPLWH in an urban South African setting, how disclosure facilitates access to social support, and how social support enables ongoing HIV care. We identified that OPLWH draw the bulk of their support from within their family networks and that this support has important direct and indirect implications for their adherence to ART and retention in care. The results show that social support is facilitated by disclosure and that disclosure is influenced by stigma, particularly perceived stigma. Although patterns and the role of social support observed within this group of OPLWH do not seem unique to OPLWH, the gendered nature of this support and the fact that age and pre-existing vulnerability affect disclosure and support from outside of the family are novel findings. The results highlight the potential value of capitalising on social support, particularly within the family by encouraging disclosure. Thinking in terms of support at multiple levels, there is value in stimulating household HIV competency to address anticipated stigma, to support an OPLWH’s needs, but this may need to be tailored for the different life circumstances of older people, undergirding existing emotional and instrumental support, and providing additional structures to increase missing informational support. Although this paper focuses on the role of family and community in social support and stigma reduction, clinics remain an important context of social support for OPLWH, especially when family and community ties are limited or cannot be relied upon. Meeting the needs of older persons as outlined here for emotional, instrumental, and informational support, could be further buttressed through age-group-specific ART support groups and clubs so that they have access to these types of support as needed and from a variety of levels.

## Figures and Tables

**Table 1:** Representation of study participants from the two sites by sex and age, n = 23.

Characteristics	Gender		Total
	Male	Female	
Community			
Khayelitsha	8	5	13
Langa	4	6	10
Age			
50–54	4	1	5
55–59	4	4	8
60–64	3	4	7
65+	1	2	3
Relationship status			
Single	0	4	4
Widowed	0	6	6
Married	10	0	10
Separated	1	1	2
Unknown	1	0	1

## Data Availability

The dataset supporting the conclusions of this article is included in the article. Original data is available by request from the authors.
